# Genome-Wide Association Study Identifies Genomic Loci Associated With Neurotransmitter Concentration in Cattle

**DOI:** 10.3389/fgene.2020.00139

**Published:** 2020-03-27

**Authors:** Qiuming Chen, Kaixing Qu, Zhijie Ma, Jingxi Zhan, Fengwei Zhang, Jiafei Shen, Qingqing Ning, Peng Jia, Jicai Zhang, Ningbo Chen, Hong Chen, Bizhi Huang, Chuzhao Lei

**Affiliations:** ^1^Key Laboratory of Animal Genetics, Breeding and Reproduction of Shaanxi Province, College of Animal Science and Technology, Northwest A&F University, Yangling, China; ^2^Yunnan Academy of Grassland and Animal Science, Kunming, China; ^3^Academy of Animal Science and Veterinary Medicine, Qinghai University, Xining, China

**Keywords:** neurotransmitter concentration, genome-wide association study, candidate genes, *Bos taurus*, *Bos indicus*

## Abstract

Abnormal neurotransmitter concentration is one of the factors that affect the health status, behavioral personality, and welfare level of animals, but the genetic basis of the abnormality is still largely unknown. The objective of this study is to identify putative genomic loci associated with neurotransmitter concentration in cattle. We measured serotonin (5HT), dopamine (DA), cortisol, glutamate (Glu), and ACTH concentrations in blood serum using double-antibody sandwich ELISA in 30 Brahman cattle and 127 Yunling cattle. Interestingly, we found that ACTH concentration was positively correlated with body weight, cannon circumference, and hip width (*P* < 0.05). Genome-wide association study (GWAS) was performed with mixed linear models using autosomal SNPs derived from the whole-genome sequence. We identified five, five, two, three, and five suggestive loci associated with 5HT, DA, cortisol, Glu, and ACTH concentration, respectively. These 20 associated loci implicated 18 candidate genes. For Glu concentration, the most significant association locus was assigned to *MCHR1*, a G-coupled receptor that could modulate glutamate release. For dopamine concentration, a very strong association locus was located in the intron of *SLC18A2*, which is a critical mediator of dopamine dynamics. However, for ACTH concentration, a very strong association locus was assigned to *HTR1F*, a G protein-coupled receptor that can influence the release of ACTH. Other candidate genes of interest identified for neurotransmitter concentration were *PRMT6*, *GADD45A*, *PCCA*, *ANGPT1*, *ACCS*, *LOC100336971*, *TNR*, *GSDMA*, *CNTN3*, *CARMIL1*, *CDKAL1*, *RBFOX1*, *PCDH15*, and *LGALS12*. Our findings will provide targets for the genetic improvement of neurotransmitter-related traits in domestic cattle and basic materials for studying the mechanism of neurotransmitter synthesis, release, and transport in human and animals.

## Introduction

Neurotransmitters are endogenous chemical substances that act as chemical messengers during synaptic transmission. Abnormal of neurotransmitter concentration predisposes to psychiatric and neurodegenerative disease in human. For example, after treatment with clomipramine, the whole-blood serotonin (5HT) content decreased in patients with obsessive-compulsive disorder (Hanna et al., 1993). In the early stages of Parkinson’s disease, the dopamine content was reduced in peripheral blood lymphocytes (Caronti et al., 1999). Therefore, investigation of neurotransmitter content in blood is helpful for understanding the mechanism of psychiatric and neurodegenerative disease in human.

In farm animals, neurotransmitter content in blood was often acted as an indicator correlating with physiological state, temperamental difference, and welfare level. In adult cow, the cortisol concentration in plasma increased after machine milking (Negrao et al., 2004). Previous behavioral experiments have demonstrated that excitable cattle exhibited higher cortisol concentration in plasma than moderate cattle (Curley et al., 2006; Cooke et al., 2019). In animal welfare, previous study has proved that minor corral changes and the adoption of good handling practices in Nellore cows can reduce the cortisol release of individuals (Lima et al., 2018). Experiments on slaughter and transportation have also demonstrated that the elevation of ACTH concentration in plasma is a response to physiological stress in cattle (Knights and Smith, 2007; Zulkifli et al., 2014). Although the revelation of genetic mechanisms underlying plasma neurotransmitter concentration will provide a genetic method to select individuals with a stable physiological state and moderate temperament to raise the welfare level and improve production efficiency in cattle, there are few studies that establish the links between neurotransmitter concentration and genetic variants.

Currently, with the decrease of sequencing cost, tens or hundreds of thousands of SNPs can be used to identify the relationship between important differential traits and genetic variants. In milk traits, a genome-wide association study (GWAS) proved that *DGAT1* and *SCD1* could affect fatty acid synthesis (Li et al., 2015). In temperament traits related to neurotransmitter concentration, previous studies have identified numerous QTLs explaining phenotypic difference (Valente et al., 2016; dos Santos et al., 2017). In fact, the number of SNPs for GWAS has risen to tens of millions. For example, based on 25.4 million imputed whole-genome sequence (WGS) variants, a meta-analysis of GWAS identified 163 genomic regions significantly associated with stature (Bouwman et al., 2018). However, no attempts have been made to identify the genomic loci associated with neurotransmitter concentrations.

The Yunling cattle breed considered in this study is a composite of 1/2 Brahman cattle (*Bos indicus*), 1/4 Murray Grey cattle (*Bos taurus*), and 1/4 Yunnan indigenous cattle (*Bos taurus* × *Bos indicus*), respectively. Therefore, it is an excellent model for the identification of genomic loci explaining important differential phenotypic traits in domestic cattle. Here, we detected five neurotransmitter concentrations in blood serum using double-antibody sandwich ELISA in 29 Brahman cattle and 128 Yunling cattle and evaluated the effect of neurotransmitter concentrations on body measurement traits. We uncovered the genetic architecture for neurotransmitter concentration by performing GWAS using the whole-genome sequence. The findings provide genomic material for genetic improvement of neurotransmitter-related traits in domestic cattle and basic materials for exploring the mechanism of neurotransmitter synthesis, release, and transport in mammals.

## Materials and Methods

### Animals

The dataset came from Brahman cattle and Yunling cattle. All test individuals are multiparous cows. To ensure the consistency of reproductive status, the subjects were not within 2 weeks pre-calving, calving, or within 2 weeks post-calving. In terms of feeding management, the experimental animals consist of 36 pen-feeding individuals (five Brahman cattle and 31 Yunling cattle) and 121 free-grazing individuals (25 Brahman cattle and 96 Yunling cattle). All pen-feeding individuals are fed a total mixed ratio (TMR) of 65% coarse and 35% concentrated fodder. From June to November every year, the free-grazing individuals eat grass in the meadow, and from December to May every year, this is supplemented with proper TMR.

### Phenotypic Analysis

We encouraged the test individuals into a squeeze crush to perform body measurement and to collect blood and tissue samples. First, we determined 15 body measurement traits using a measuring tape and a measuring stick. These 15 body measurement traits comprised the withers height, hip cross height, body length, chest circumference, abdominal circumference, cannon circumference, chest width, chest depth, hip circumference, hip width, ischium width, head length, forehead size, rump length, and body length. Next, the whole blood was collected from a jugular vein to detect neurotransmitter concentration. Finally, ear tissue was collected to extract genomic DNA using an ear punch.

Serum was harvested from centrifuged whole blood samples (2000 g centrifugation for 20 min) and then stored at −80°C prior to neurotransmitter concentration determination. Neurotransmitter concentrations were determined by the double-antibody sandwich ELISA method according to the manufacturer’s manuals (MMBio, Jiangsu, China) by comparison of samples with standard curves generated with known concentrations. The absorbance was originally obtained from a Multiskan MS Primary EIA V. 1.5-0 reader at a wavelength of 450 nm. In addition, we checked for the effect of breed and feeding regime on neurotransmitter concentration using the general linear model in R software (“lm” function).

### Genome Sequencing, Alignment, and SNP Detection

DNA extracted from ear tissues taken from Brahman cattle and Yunling cattle was used for this analysis. The standard phenol-chloroform protocol was used to isolate DNA (Green and Sambrook, 2012). Whole-genome sequencing was performed using the Illumina NovaSeq platform. The size of the insert fragment was 500 bp. Finally, ∼16.51 billion reads were generated. Pair-end sequence reads were mapped to the reference *Bos taurus* genome (ARS-UCD1.2) using BWA-MEM (Li and Durbin, 2009) with default parameters. Picard was used to exclude potential duplicate reads (“REMOVE_DUPLICATES = true”). We used the Genome Analysis Toolkit 3.8 (GATK) (Nekrutenko and Taylor, 2012) (“HaplotypeCaller,” “GenotypeGVCFs,” and “SelectVariants” modules) to call candidate SNPs. To filter SNPs and avoid possible false positives, the “VariantFiltration” module of GATK was adopted with the following options: (1) SNPs with QD (variant confidence/quality by depth) < 2 were filtered; (2) SNPs with FS (Phred-scaled *P*-value using Fisher’s exact test) > 60 were filtered; (3) SNPs with Mapping Quality Rank Sum < −12.5 were filtered; (4) SNPs with Read Pos RankSum < −8.0 were filtered; (5) SNPs with sequence depth (for all individuals) < 1/3x or >3x were filtered. Inference of haplotype phase and imputing of missing alleles were performed using Beagle (Browning and Browning, 2007). In addition, we performed principal component analysis using smartPCA in the EIGENSOFT v5.0 package (Patterson et al., 2006) to adjust population stratification.

#### Partial Correlation Analysis

To investigate the relationship between neurotransmitter concentrations and body measurement traits, partial Pearson’s correlation adjusting for ancestry (principal component 1), feeding regime, and breed was computed using the ppcor package (Kim, 2015) in R.

### GWAS Analysis

After filtering the SNPs with MAF > 0.1 or missing rate > 0.1, a total of 12983056 SNPs remained and were used to carry out GWAS analysis for neurotransmitter concentrations. The association analysis was carried out using the Genome-Wide Efficient Mixed-Model Association (GEMMA) software package (Zhou and Stephens, 2012). The mixed linear model assumed the following model:

y=Xα+Sβ+Kμ+ε

where *y* is a vector of phenotypes, α is a vector of fixed effects representing marker effects, β is a vector of fixed effects representing non-marker effects, and μ is a vector of unknown random effect. *X*, *S*, and *K* represent the incidence matrices related to α, β, and μ, respectively, and ε represents a vector of random residual effects. The principal component 1 was defined as the *S* matrix. The kinship matrix calculated from nucleotide polymorphism was defined as the *K* matrix.

To estimate the correction required for multiple testing, the SNP data was subsequently pruned for linkage disequilibrium in PLINK (Purcell et al., 2007), using a 50 SNP sliding window with a five SNP increment between windows, retaining only SNPs with a pairwise *r*^2^ < 0.2. The number of LD-pruned SNPs (747,835) was defined as the effective number of independent SNPs. Therefore, the *P*-value thresholds were set at 6.7 × 10^–8^ (significant, 0.05/747835) and 1.3 × 10^–6^ (suggestive, 1/747835).

After completing GWAS, we further narrowed down our findings to obtain corresponding candidate genes. Firstly, we calculated the pairwise linkage disequilibrium among SNPs associated with neurotransmitter concentration. The borders of associated loci were defined according to the LD value (*r*^2^ > 0.6). False-positive signals were filtered for by retaining the associated loci with ≥2 suggestive SNPs. The LD plots of suggestive SNPs for associated loci were visualized using the Haploview program (Barrett et al., 2005). Secondly, the SNP with the smallest *P*_*wa*__*l*__*d*_ value in an association locus with neurotransmitter concentrations was defined as the leading SNP. Finally, we performed functional annotation for suggestive SNPs associated with neurotransmitter concentrations using ANNOVAR (Wang et al., 2010) according to the *Bos taurus* reference genome (ARS-UCD1.2).

## Results

### Effect of Neurotransmitter Concentration on Body Measurement Traits

We measured five neurotransmitter concentrations, including serotonin (5HT), dopamine (DA), cortisol, glutamate (Glu), and ACTH in blood serum of Brahman cattle and Yunling cattle using the double-antibody sandwich ELISA method. The descriptive statistics of the five neurotransmitter concentrations are summarized in Table 1. The coefficients of variation of 5HT, DA, cortisol, Glu, and ACTH concentrations were 19.81, 18.42, 17.45, 17.23, and 18.87%, respectively.

**TABLE 1:** Descriptive statistics of five neurotransmitter concentrations.

Trait	Maximum	Minimum	Mean	*SD*	CV (%)	Skewness	Kurtosis
5HT (ng/L)	3786	1948	2844	563.53	19.81	0.067	1.63
DA (ng/L)	228.4	112.6	168.3	31.01	18.42	0.164	1.89
Glu (μmol/L)	43.95	24.08	33.76	5.89	17.45	0.070	1.70
Cortisol (μg/L)	228.2	116.7	172.0	29.63	17.23	−0.001	1.86
ACTH (μg/L)	55.90	28.49	41.60	7.85	18.87	0.200	1.87

**FIGURE 1:**
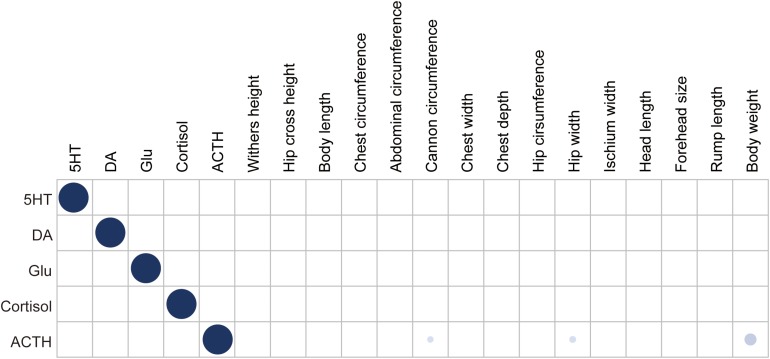
Heatmap depicting Pearson’s correlation coefficients between neurotransmitter concentration and body measurement traits. Dot size and color saturation represent the strength of the correlation. Significant correlations (*P* ≤ 0.05) are marked in blue.

We used a general linear model to test the influence of breed and feeding regime on the five neurotransmitter concentrations. The result showed that there was no significant difference between Brahman cattle and Yunling cattle or between free grazing and pen feeding. We also calculated the pairwise partial correlations among neurotransmitter concentrations (Figure 1). The result showed that there was no significant correlation among neurotransmitter concentrations. Meanwhile, the association between neurotransmitter concentrations and the 15 body measurement traits was also assessed using the above approach (Figure 1). It is remarkable that only ACTH concentration was positively correlated with body weight (*r*_*s*_ = 0.251, *P* = 3.31 × 10^–3^), cannon circumference (*r*_*s*_ = 0.163, *P* = 0.043), and hip width (*r*_*s*_ = 0.168, *P* = 0.038).

### Whole-Genome Data Description

**FIGURE 2:**
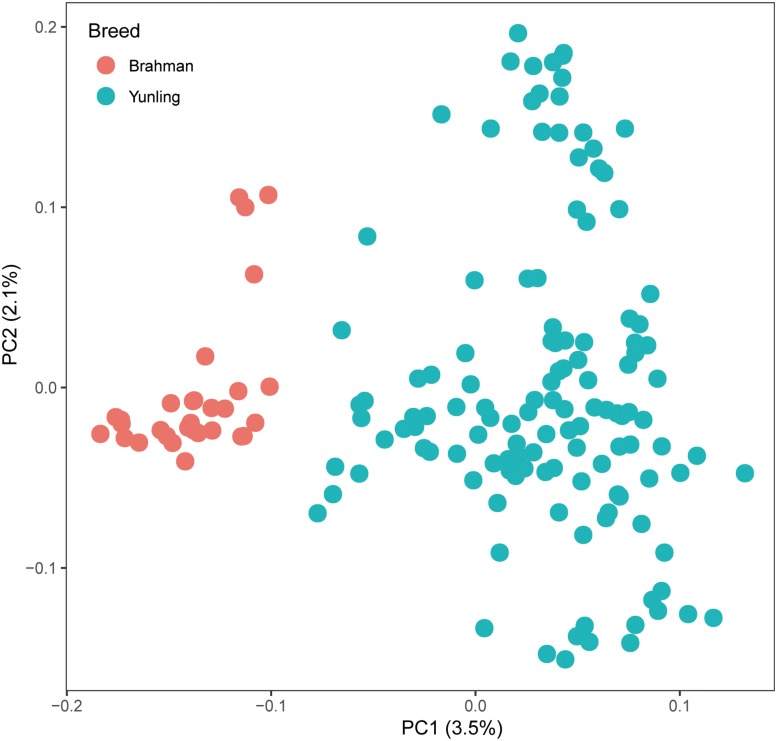
PC plot (PC1 against PC2) using autosomal SNP markers.

Genomic DNA samples from 157 individuals were sequenced to ∼5.60 × genome coverage each. About 16.51 billion reads were aligned to the *Bos taurus* reference genome sequence ARS-UCD1.2, and the average alignment rate was 99.55% (Supplementary Table S1). After filtering raw SNPs, a total of ∼40.98 million SNPs were retained. In principal component analysis, principal component 1 explained 3.5% of the total variation and separated Brahman cattle from Yunling cattle (Figure 2).

### Genome-Wide Association Studies for Five Neurotransmitter Concentrations

**FIGURE 3:**
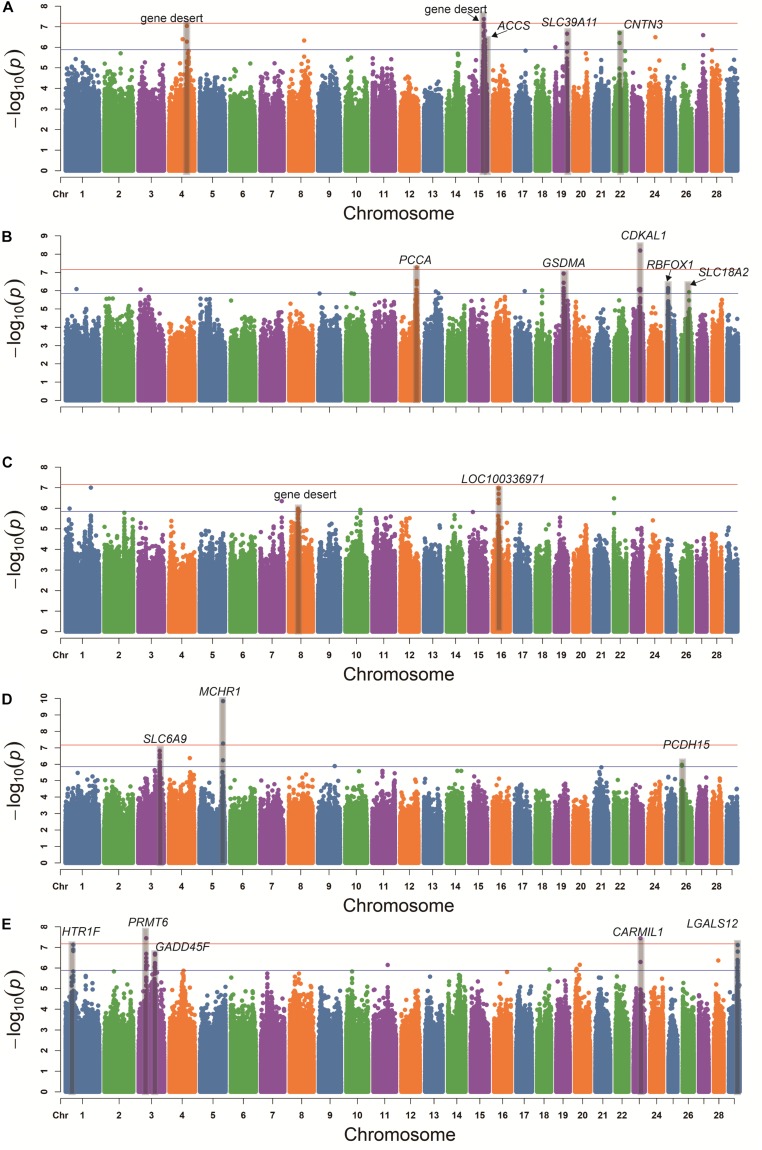
Genome-wide association study of the concentrations of 5HT **(A)**, DA **(B)**, cortisol **(C)**, Glu **(D),** and ACTH **(E)** in blood serum using the mixed linear model. Red line and blue line indicate the significant threshold and suggestive threshold, respectively.

The GWAS Manhattan plot for 5HT concentration in blood serum is shown in Figure 3A. In total, 36 SNPs were found to be suggestively associated with variation in 5HT concentration (Supplementary Table S2). After detecting the linkage disequilibrium of these SNPs, we identified five suggestive association loci for 5HT concentration in blood serum (Table 2). According to the annotation of ANNOVAR software, the most significant locus was observed on BTA15 and was located in gene desert (regions over 500 kb that are devoid of protein-coding genes). The second-ranked locus was observed on BTA4 and was also located in gene desert. The third-ranked locus was observed on BTA15, and its leading SNP was located in the intron of *ACCS*. The remaining association loci were observed on BTA19 and BTA22, and their candidate genes embodied *SLC39A11* and *CNTN3*, respectively.

**TABLE 2:** Descriptive summary of GWAS for five neurotransmitter concentrations.

Associated locus	Leading variant	MAF	-log_10_*P*_*wald*_	Trait	Candidate gene
1:35945547–35945584	1:35945558	0.236	7.13	ACTH	*HTR1F*
3:36525172–36526078	3:36526016	0.229	7.45	ACTH	*PRMT6*
3:77705305–77728559	3:77728559	0.398	6.70	ACTH	*GADD45A*
3:101918733–102495677	3:101918733	0.382	6.80	Glu	*SLC6A9*
4:84001170–84005564	4:84005564	0.490	7.04	5HT	Gene desert
5:111980228–111980732	5:111980732	0.242	9.84	Glu	*MCHR1*
8:42958091–42963716	8:42963716	0.481	5.97	Cortisol	Gene desert
12:76815037–76912606	12:76859881	0.223	7.28	DA	*PCCA*
15:68085551–69989675	15:69208676	0.277	7.37	5HT	Gene desert
15:74154782–74195072	15:74186549	0.143	6.80	5HT	*ACCS*
16:28978311–28979773	16:28978311	0.299	7.01	Cortisol	*LOC100336971*
19:40301775–40310333	19:40301775	0.484	6.93	DA	*GSDMA*
19:58424044–58426162	19:58424061	0.487	6.66	5HT	*SLC39A11*
22:27517768–27527029	22:27517768	0.347	6.69	5HT	*CNTN3*
23:32343204–32343711	23:32343711	0.178	7.45	ACTH	*CARMIL1*
23:37336886–37344450	23:37344450	0.255	8.19	DA	*CDKAL1*
25:5800850–5848346	25:5800850	0.169	6.13	DA	*RBFOX1*
26:5310758–5310786	26:5310758	0.223	5.96	Glu	*PCDH15*
26:37563598–37563603	26:37563603	0.182	5.92	DA	*SLC18A2*
29:41276440–41791032	29:41791032	0.172	7.11	ACTH	*LGALS12*

In Figure 3B, the GWAS for DA concentration shows that 25 SNPs were found to be suggestively associated with the variation of DA concentration (Supplementary Table S3). After detecting the linkage disequilibrium of these SNPs, we identified five suggestive association loci for DA concentration (Table 2). Based on the annotation of ANNOVAR software, the most significant locus was observed on BTA23 and was located in the intron of *CDKAL1*. The second-ranked locus, which was located in the intron of *PCCA*, was observed on BTA12. The third-ranked locus was observed on BTA19, and its leading SNP was located in the intron of *GSDMA*. The remaining association loci were observed on BTA25 and BTA26, and their candidate genes included *RBFOX1* and *SLC18A2*, respectively.

Figure 3C shows the GWAS Manhattan plot for cortisol concentration in blood serum. In total, 12 SNPs were found to be suggestively associated with variation in cortisol concentration (Supplementary Table S4). After detecting the linkage disequilibrium of these SNPs, we identified two suggestive association loci for cortisol concentration (Table 2). After annotation using ANNOVAR, the most significant locus was observed on BTA16, and its nearest gene was *LOC100336971*. Another associated locus was observed on BTA8 and was located in gene desert.

The GWAS Manhattan plot for Glu concentration in blood serum is shown in Figure 3D. In total, 11 SNPs were found to be suggestively associated with variation of Glu concentration (Supplementary Table S5). After detecting the linkage disequilibrium of these SNPs, we identified three suggestive association loci with Glu concentration (Table 2). By means of ANNOVAR annotation, we found that the most significant locus was observed on BTA5 and was located ∼8 Kb upstream of *MCHR1*. The second-ranked locus was observed on BTA3, and its leading SNP was located upstream of *KLF17*. The third-ranked locus was observed on BTA26 and located in the intron of *PCDH15*.

In Figure 3E, our result shows that 34 SNPs were found to be suggestively associated with the variation in ACTH concentration in blood serum (Supplementary Table S6). After detecting the linkage disequilibrium of these SNPs, we identified five suggestive association loci for ACTH concentration (Table 2). The most significant locus was observed on BTA3 and was located downstream of *PRMT6*. The second-ranked locus was observed on BTA23 and was located in the intron of *CARMIL1*. The third-ranked locus was observed on BTA1, and its leading SNP was located upstream of *HTR1F*. The remaining associated loci were observed on BTA3 and BTA29, and their candidate gene contained *GADD45A* and *LGALS12*, respectively.

**FIGURE 4:**
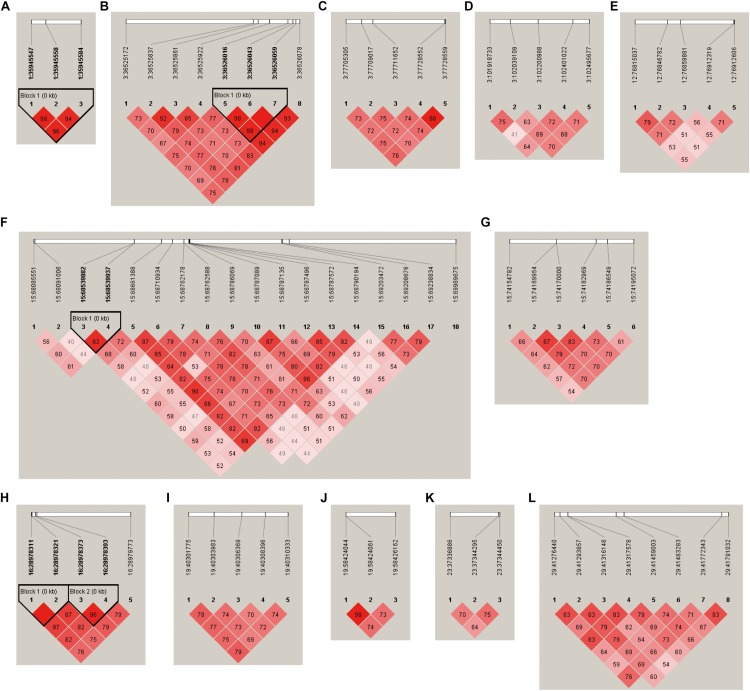
LD plots of suggestive SNPs on neurotransmitter concentration at the **(A)** 1:35945547–35945584 locus, **(B)** 3:36525172–36526078 locus, **(C)** 3:77705305–77728559 locus, **(D)** 3:101918733–102495677 locus, **(E)** 12:76815037–76912606 locus, **(F)** 15:68085551–69989675 locus, **(G)** 15:74154782–74195072 locus, **(H)** 16:28978311–28979773 locus, **(I)** 19:40301775–40310333 locus, **(J)** 19:58424044–58426162 locus, **(K)** 23:37336886–37344450 locus, and **(L)** 29:41276440–41791032 locus.

The LD plots of 12 loci at which the number of suggestive SNPs associated with neurotransmitter concentration in blood serum was above 3 are presented in Figure 4. The results show that four loci had LD blocks (the 1:35945547-35945584 locus, 3:36525172-36526078 locus, 15:68085551-69989675 locus, and 16:28978311-28979773 locus) with a length of less than 1 kb. There were no LD blocks at the remaining eight loci.

## Discussion

Previous studies on neurotransmitter concentration in blood have primarily focused on physiological difference under different conditions (Knights and Smith, 2007; Zulkifli et al., 2014; Lima et al., 2018) or analysis of its correlation with temperament in cattle (Curley et al., 2006; Cooke et al., 2019). In our present study, cattle with a higher ACTH concentration in blood serum tended to have better stature for production (i.e., higher body weight, higher cannon circumference, higher hip width). To our knowledge, this is the first correlative identification between ACTH concentration and body measurement traits. Although the correlation is only phenotypic, the positive correlation between ACTH concentration and body measurement traits suggests that improvement of ACTH concentration will have a positive impact on cattle production.

Subsequently, we implemented GWAS to identify the genomic loci explaining the phenotypic variance in neurotransmitter concentrations in blood serum using the whole-genome sequence. We identified five, five, two, three, and five suggestive loci associated with 5HT, DA, cortisol, Glu, and ACTH, respectively, suggesting that neurotransmitter concentration in blood serum is polygenetically controlled. To our knowledge, this is the first identification of genomic loci associated with neurotransmitter concentration in blood serum in cattle using GWAS. Moreover, we found that the LD level for the suggestive loci was very low, suggesting that the loci associated with neurotransmitter concentration were not the target of phenotypic selection or did not experience bottlenecks or gene drift. Although our sample size was smaller for GWAS, the variants with high frequency and large effect have been identified, and further GWAS with a larger sample size will result in the identification of additional variants with low frequency and small effect in future. In addition, our genome coverage was very low (∼5.60×), but a previous study has shown that very low-depth whole-genome sequencing is an efficient alternative to complex trait association studies (Schwartzentruber et al., 2018).

Among 20 suggestive loci, the most significant locus was associated with glutamate concentration in blood serum and was located 8 kb upstream of *MCHR1*, a G-coupled receptor for the neuropeptide melanin-concentrating hormone, which modulates glutamate release from presynaptic terminal (Gao and van den Pol, 2001). Knockout *MCHR1* mice exhibited reduced anxiety-like behavior (Roy et al., 2006). Another strong locus associated with glutamate concentration was assigned to *SLC6A9*, encoding a glycine transporter. In previous studies, glycine has been found to act as an inhibitory neurotransmitter in the central nervous system (Alfadhel et al., 2016) and an obligatory co-agonist of glutamate involved in the regulation of glutamatergic neurotransmission (Johnson and Ascher, 1987), which suggested that *SLC6A9* may participate in the glutamate transporter. For dopamine concentration in blood serum, a strong locus was located in the intron of *SLC18A2*, which is a critical mediator of dopamine dynamics in neuronal terminal (Lohr et al., 2015). Another strong locus associated with dopamine concentration was located in the intron of *PCCA*, which is the alpha subunit of the heterodimeric mitochondrial enzyme Propionyl-CoA carboxylase (Stankovics and Ledley, 1993). It has been demonstrated that dopa decarboxylase is involved in dopamine synthesis (Seifert et al., 1980), suggesting that *PCCA* may participate in the dopamine synthesis. In terms of ACTH concentration, the nearest gene of a leading SNP was *HTR1F*, a G protein-coupled receptor for 5HT, which can regulate the HPA axis by moderating the output of corticotropin-releasing hormone in the hypothalamus and further influence release of ACTH (Falkenberg and Rajeevan, 2010). The identification of these candidate genes may provide better opportunities for investigating the molecular mechanism of neurotransmitter synthesis, release, and transport in mammals.

In conclusion, our study revealed that ACTH concentration in blood serum was significantly related to body measurement traits (body weight, cannon circumference, hip cross height, and hip width). We performed GWAS for neurotransmitter concentration in blood serum using autosomal SNPs derived from WGS and then identified five, five, two, three, and five suggestive loci associated with 5HT, DA, cortisol, Glu, and ACTH, respectively. These 20 loci implicated 17 candidate genes, including *MCHR1* (a G-coupled receptor involved in glutamate release), *SLC18A2* (a critical mediator of dopamine dynamics), and *HTR1F* (a G protein-coupled receptor involved in release of ACTH). The revelation of the genetic underpinnings of neurotransmitter concentration will provide theoretical guidance for the improvement of neurotransmitter concentration by genetic manipulation to reduce stress, elevate welfare level, and boost productivity in cattle. In addition, our findings are helpful for follow-up studies to identify causal variants of difference in neurotransmitter concentration in blood serum and investigate the molecular mechanism of neurotransmitter synthesis, release, and transport in mammals.

## Data Availability Statement

Sequences are available from GenBank with the BioProject accession number PRJNA555741.

## Ethics Statement

This study was approved by the Institutional Animal Care and Use Committee of Northwest A&F University (Permit Number: NWAFAC1019).

## Author Contributions

CL and BH conceived and designed the project. QC, KQ, JZ, QN, PJ, JZ, NC, and HC carried out sampling. QC, FZ, and JS performed the analyses. QC wrote the manuscript. ZM revised the manuscript. All authors reviewed and approved the final manuscript.

## Conflict of Interest

The authors declare that the research was conducted in the absence of any commercial or financial relationships that could be construed as a potential conflict of interest.

## Supplementary Material

TABLE S1 Summary of sequencing data.

TABLE S2 The suggestive SNPs associated with 5HT concentration in blood serum.

TABLE S3 The suggestive SNPs associated with DA concentration in blood serum.

TABLE S4 The suggestive SNPs associated with cortisol concentration in blood serum.

TABLE S5 The suggestive SNPs associated with Glu concentration in blood serum.

TABLE S6 The suggestive SNPs associated with ACTH concentration in blood serum.
